# Editorial: Insights in visual neuroscience: 2023

**DOI:** 10.3389/fnins.2024.1396011

**Published:** 2024-04-15

**Authors:** Benjamin Thompson, Vallabh E. Das, Ione Fine

**Affiliations:** ^1^School of Optometry and Vision Science, University of Waterloo, Waterloo, ON, Canada; ^2^Centre for Eye and Vision Research Ltd., Hong Kong, Hong Kong SAR, China; ^3^Liggins Institute, University of Auckland, Auckland, New Zealand; ^4^College of Optometry, University of Houston, Houston, TX, United States; ^5^Department of Psychology, University of Washington, Seattle, WA, United States; ^6^Center for Human Neuroscience, University of Washington, Seattle, WA, United States; ^7^School of Biomedical Sciences, Faculty of Biological Sciences, University of Leeds, Leeds, United Kingdom

**Keywords:** vision, visual development, entoptic phenomena, amblyopia, color vision, neuroplasticity

Visual neuroscience is an inherently interdisciplinary field spanning biology, physiology, physics, psychology, clinical practice, and more. The knowledge gained from advances in visual neuroscience not only deepens our understanding of the complex mechanisms that allow us to see, but also has practical applications across a wide range of areas including eye and vision health, telecommunications, and computer vision. The review papers in this Research Topic reflect the breadth and depth of visual neuroscience research. The topics covered illustrate the vast array of topics encompassed in visual neuroscience, including the fundamental neural mechanisms that support vision—from genetics and photopigments to cortical plasticity.

Approximately 5% of males have a color vision deficiency (dyschromatopsia). With sophisticated genetic analyses, it is now possible to understand the full complexity of dyschromatopsia. Yang et al. provide a comprehensive review of the mechanisms that support human color vision and how these mechanisms are altered in individuals with dyschromatopsia. The authors also review research into the recovery of color vision, ranging from gene therapy to optical filters. Although current treatments for dyschromatopsia are either at a preclinical stage or of questionable efficacy, the authors strike a positive note for the future suggesting that effective clinically proven treatments may emerge.

Polarization is a fundamental property of light that is utilized by the visual systems of birds, insects, fish and other marine creatures. Although the human visual system has not evolved to utilize polarization directly, the structure of the macular pigment in the Henley fiber layer of the retina acts as a radial filter for blue light that enables the perception of entoptic images created by polarization. Haidinger's brush is a well-known entopic phenomenon that has been directly linked to the radial polarizer in the human eye. In the third review of this Research Topic, Pushin et al. describe the development of a structured light source with spatially varying polarization profiles that they have used to explore the perception of polarization by humans. The team are now able to produce highly visible entoptic images with structured light. Because the images represent a precise interaction between the light source and the structure of the retina, Pushin et al. are now investigating the use of structured light induced entoptic images to detect the very earliest stages of macular degeneration.

Many studies of the neural circuitry supporting vision focus on visually responsive regions of the cerebral cortex. However, the thalamus is a key component of the visual pathway. Casanova and Chalupa describe how the lateral geniculate nucleus (LGN) and the pulvinar within the thalamus play critical roles in all aspects of visual cognition and explain the importance of continuing to study the role of these structures in visual perception. After providing a detailed review of what is currently known about feedforward and feedback processing in the LGN and pulvinar, Casanova and Chalupa highlight the way in which emerging neuroscientific techniques such as optogenetics, connectomics and ultra-high resolution functional magnetic resonance imaging may enable the next generation of visual neuroscientists to advance the field.

Depth perception, referred to as stereopsis, supports hand-eye coordination, walking and balance. In her perspective article on peripheral stereopsis, Verghese argues for the importance of depth perception in peripheral vision, particularly in disorders such as macular degeneration and amblyopia where central visual function is impaired in one or both eyes. Importantly, Verghese indicates that peripheral depth perception can be enhanced with training, opening the possibility for more successful recovery of functional vision in patients with central vision loss.

Amblyopia is a leading cause of monocular visual impairment in children and adults. Deprivation of sharp vision in the 1^st^ months of life, often due to unilateral congenital cataract, induces the most severe type of amblyopia, monocular deprivation amblyopia (MD). Duffy et al. provide a comprehensive review of the insights gained from decades of work in cat, monkey and rodent models using monocular deprivation, and identify strengths and limitations of working with each of these model systems. They articulate a case for why research using animal models for amblyopia is critical by laying out how insights from animal research have led to formulation of promising new and innovative therapies.

Perhaps the capacity that most differentiates humans from other animals is their ability to rapidly learn novel skills over development. Many of these tasks involve the development of novel specialized areas in the brain or “hyper-abilities” within existing areas. Park and Fine highlight commonalities between the sensory deprivation and skill acquisition literature and suggest that shared functional, algorithmic, and structural constraints might mediate both types plasticity. Merging these two bodies of research has the potential to provide important new insights into the extraordinary flexibility of human cognitive development.

Non-invasive brain stimulation is an emerging technology that enables modulation of neurotransmitter concentrations and neural excitability in targeted, superficial areas of the cerebral cortex such as the primary visual cortex. Bello et al. report the results of a structured review and meta-analysis of studies exploring whether non-invasive brain stimulation can enhance normal vision. The results indicate that some fundamental visual functions such as contrast sensitivity can indeed be improved, but that new, well-designed, and appropriately powered studies will be required to identify optimal stimulation parameters and the range of visual abilities that can be enhanced.

Together, the reviews and perspectives presented in this Research Topic highlight breakthroughs that have advanced visual neuroscience and identify promising pathways where this field is likely to make rapid progress. However, there is still much to be learned. As highlighted in the closely related Research Topic—Rising Stars in Visual Neuroscience, the next generation of researchers are taking on this important challenge.

## Author contributions

BT: Conceptualization, Writing – original draft. VD: Conceptualization, Writing – review & editing. IF: Conceptualization, Writing – review & editing.

